# Personalized Feedback for Personalized Trials: Construction of Summary Reports for Participants in a Series of Personalized Trials for Chronic Lower Back Pain

**DOI:** 10.1162/99608f92.d5b57784

**Published:** 2022-09-08

**Authors:** Stefani D’Angelo, Heejoon Ahn, Danielle Miller, Rachel Monane, Mark Butler

**Affiliations:** Institute of Health System Science, Feinstein Institutes for Medical Research, Northwell Health; Manhasset, NY, USA

**Keywords:** patient education, lay summary, patient feedback, personalized trial, lower back pain, virtual, patient report

## Abstract

Personalized (N-of-1) trials offer a patient-centered research approach that can provide important clinical information for patients when selecting which treatment options best manage their chronic health concern. Researchers utilizing this approach should present trial results to patients in a clear and understandable manner in order for personalized research trials to be useful to participants. The current study provides participant feedback examples for personalized trial reports using lay summaries and multiple presentation styles from a series of 60 randomized personalized trials examining the effects of massage and yoga versus usual care on chronic lower back pain (CLBP). Researchers generated summary participant reports that describe individual participant results using multiple presentation modalities of data (e.g., visual, written, and auditory) to offer the most appealing style for various participants. The article discusses contents of the participant report as well as participant satisfaction with the personalized summary report, captured using a satisfaction survey administered after study completion. The results from the satisfaction survey in the current study show that participants were generally satisfied with their personalized summary report. Researchers will use feedback from the participants in the current study to refine personalized feedback reports for future studies.

## 1. Introduction

The presentation of trial results to research participants and a lay audience is an important aspect of science; interventions must be acceptable to both clinicians and patients in order to become ubiquitous in practice. However, researchers must present information to the public regarding the results of research trials in a manner that is precise and clear with minimal potential for misunderstanding. Misinterpretation of results by the general public has led to scientific misconceptions and public health issues that are still being dealt with today (Eggertson, 2010)(Fihn, 2019)(Gonon et al., 2011)(Mehr, 2015). The increasing importance of dissemination of trial results to the general public and study participants has led to the development of guidelines for verbal and visual dissemination of results (Bennett, 2005)(n.d.-a). Researchers create ‘lay summaries’ for trial results presented verbally to nonscientific audiences. Lay summaries are descriptions of the design and results of a trial that use ‘plain language’ and are developed to be understandable by participants at common reading levels (Barnes & Patrick, 2019). Essential components of lay summaries include the study description, research questions, description of the treatment(s), conditions being treated, results of the study, adverse events, and how the trial will be able to help the participant and others in the future (Barnes & Patrick, 2019)(n.d.-b)(n.d.-c). A well-articulated lay summary should be able to provide a research participant and a member of the general public with an understanding of the basics of the study, the results that were found, and why the study matters (Barnes & Patrick, 2019)(n.d.-d). Presentation of lay summaries should avoid the use of any jargon. When presenting results visually, researchers should clearly present informative results without visual distractions (n.d.-e). Visual results should not take the form of marketing materials but should rather reflect “something a clinical investigator might draw to illustrate the results of a study in a conversation with a patient” (n.d.-f). These standards for presentation of results are usually applied to large clinical trials where multiple participants engage in the same intervention. As a result, lay summaries usually involve presentation of aggregate data across all participants in a trial and are not necessarily formatted for individual patients or research participants.

Personalized trials are a patient-centered research approach that can provide important clinical information for patients in selecting which treatments work best to manage their health condition. A personalized trial design involves assessing individual patients using multiple crossover trials with alternating time periods of treatment, alternative treatment, and placebo therapies in randomized blocks (G. Guyatt, 2016)(G. H. Guyatt et al., 1990). During a personalized trial, objective data recorded from wearable devices, often continuously throughout the trial, supplement self-report data collected from the participant multiple times each day. Personalized trials specifically use this data to help patients make health care decisions informed by high-integrity, evidence-based information uniquely relevant to the outcomes and values important to them (Duan et al., 2013). Prior series of personalized trials led participants to opt to change treatment, cease treatment, or confirm the initial treatment (Duan et al., 2013)(G. Guyatt, 2016)(G. H. Guyatt et al., 1990)(Joy et al., 2014)(Larson, 2010). Despite the utility of personalized trials at the patient level, clinicians seldomly use personalized designs in clinical practice (Gabler et al., 2011)(G. Guyatt, 2016)(Kravitz et al., 2008)(n.d.-g).

The focus of personalized designs on individual patient treatment should also lead to a tailored presentation of trial results for patient use. However, researchers have traditionally initiated personalized trials with clinicians—rather than patients—as the target audience for results (Kravitz et al., 2008). Consequently, personalized trial administrators tend to report results using standardized scientific guidelines (Vohra et al., 2015) without much consideration for participant understanding and interpretability. This conventional reporting includes trial results estimated as means with standard deviations or confidence intervals as well as measures of statistical significance for comparisons between treatments. While the established guidelines provide valuable information for personalized trial results in communicating with other researchers and clinicians, this presentation of results can often be confusing to patients (Glenton et al., 2010).

The patient education literature provides some guidance for communicating results to individual patients. This literature emphasizes the importance of taking individual patient learning styles into account when communicating information (Inott & Kennedy, 2011) as each patient may have differing preferences for how information is presented via visual, written, or auditory formats (n.d.-h). Patients’ motivation and literacy levels may also affect their learning styles (Beagley, 2011). Effective patient education and communication of medical information should include presentation of information in multiple formats to account for varying preferences in learning style while accounting for low levels of health literacy and varying levels of patient motivation (Inott & Kennedy, 2011).

The current study examines the effectiveness and acceptability of a feedback report developed during a personalized intervention to reduce chronic lower back pain (CLBP) via massage and yoga treatment. These study reports incorporate aspects of lay summaries of research findings and information from the patient education literature to ensure that essential elements of the personalized trial research findings are communicated to participants in a manner that is both informative and easy to understand. We will discuss the goals of the intervention, why communication of results was important in this patient-centered trial, how individualized participant reports were generated, and how we tried to incorporate guidelines for presenting trial findings in our individual patient reports. We will also report participant satisfaction levels and feedback regarding the individual reports, which will be used to refine the presentation of personalized trial results to participants in the future.

## 2. Methods

### 2.1. Study Design

The report of the primary findings from this study, entitled “A Series of Virtual Interventions for Chronic Lower Back Pain: A Feasibility Pilot Study for a Series of Personalized (N-of-1) Trials” contains full details regarding the design and methods of the trial (n.d.-i). This report focuses on personalizing trial findings for participants and presents details relevant to the feedback report.

The current study involved a series of 60 randomized personalized trials examining the effects of massage and yoga versus usual care on CLBP. Study participants received the intervention virtually over the course of 14 weeks in one of two possible treatment orders. The study team utilized two treatment orders to simplify implementation of the pilot while eliminating linear trends for analyses of treatment effects on pain. The trial provided participants with a Fitbit Charge 3^™^ device and encouraged participants to wear the Fitbit device at all times, including during sleep. The study also asked participants to assess their pain, stress, and fatigue three times daily via ecological momentary assessment (EMA) delivered by text message. Each evening, participants answered a survey questionnaire assessing their back pain and pain management strategies. Each weekend, participants completed a longer survey measure asking them to reflect on their pain and pain management over the course of the preceding week.

Within 3 months of study completion, researchers provided participants with a personalized report containing their analyzed data. Researchers sent a satisfaction survey to all participants within one week of the delivery of their personalized report summarizing their study results. In addition, study coordinators reached out to each participant to interview them about their experience with the personalized report following completion of their satisfaction survey. Study enrollment began in November 2019 and all study activities finished in January 2021.

#### 2.1.2. Primary and Secondary Outcomes

The primary outcome measure for the trial was the System Usability Scale (SUS; (Bangor et al., 2008). The researchers chose the SUS as a validated tool to assess the efficiency and user satisfaction with the overall personalized trials system presented, including the participant report (n.d.-j)(Lewis, 2018). Additional unvalidated questions regarding overall trial satisfaction as well as individual components of the trial were assessed for quality improvement purposes. (n.d.-k) further report these outcomes in the primary outcome article for the trial. The feedback report to participants included several secondary outcomes. The patient-relevant outcomes measured in the study included several measures of pain using PROMIS (Patient-Reported Outcomes Measurement Information System) pain scales (Amtmann et al., 2010)(Revicki et al., 2009) modified to be assessed daily rather than weekly. These measures included those used to assess intensity of pain symptoms (Pain Intensity 3a Fixed Length Short Form) and pain interference (Pain Interference 4a Fixed Length Short From) with daily life due to pain symptoms over the past 24 hours. Participants also completed EMA ratings three times daily. This assessment used the single-item Numeric Pain Rating Scale assessment administered via text message and delivered at randomized times throughout each participant’s self-reported wake and sleep windows. In addition to measures related to pain, participants completed EMA assessments of both self-reported fatigue and self-reported stress. Participants also self-reported daily use of pain medication. Clinical outcomes included objective data recorded through continuous wear of the Fitbit device. The participant report included information for each of the clinical outcomes listed above.

### 2.2. Participant Reports

The study presented participants with personalized reports summarizing their trial results and showing the effectiveness of massage and yoga treatments compared to usual care treatments for each of the clinical outcomes. The goal of the participant reports was to highlight overall levels of pain experienced by participants, identify pain levels during each of the treatment phases (i.e., yoga, massage, and usual care), show comparisons of pain between treatment and usual care phases, and identify differences in pain between trial phases that were determined to be statistically significant. The participant report presented summaries and results for additional clinical outcomes, but the effectiveness of yoga and massage treatment versus usual care for pain symptoms was the primary emphasis of the report. In addition, the participant report summarized information about his or her adherence to yoga treatment, massage treatment, and study measures.

**Figure 1.**
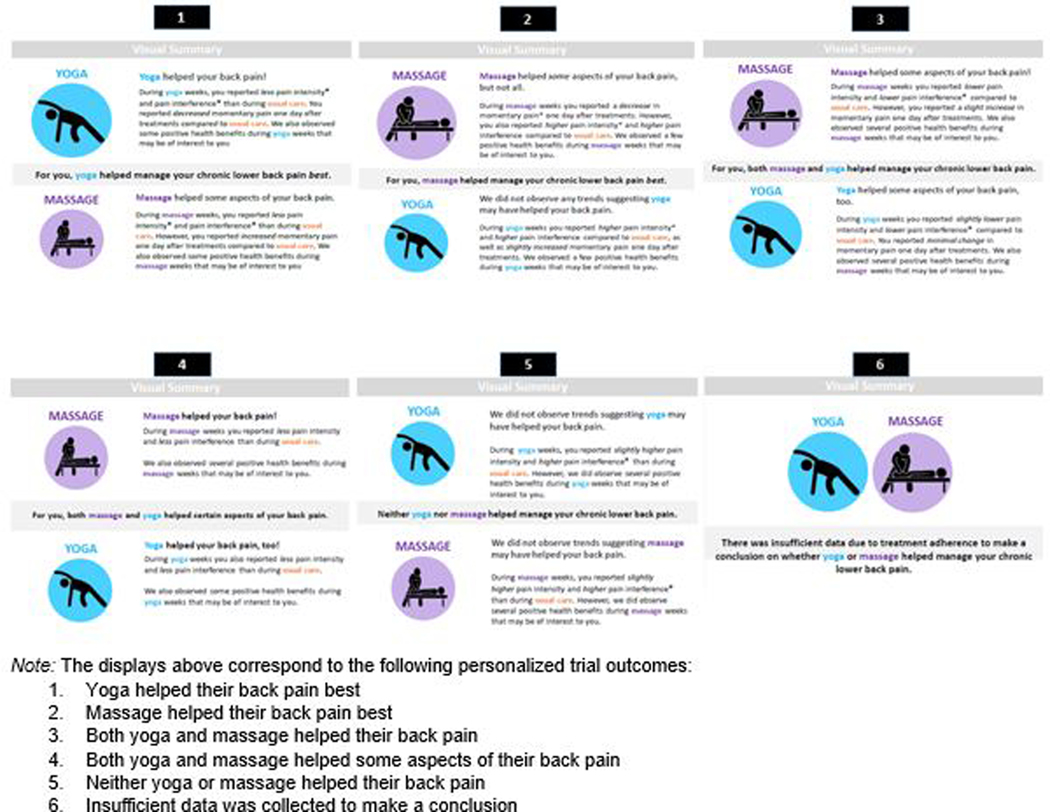
Verbal summaries of pain outcomes for the personalized trial. *Note.* The displays above correspond to the following personalized trial outcomes: 1. Yoga helped their back pain best 2. Massage helped their back pain best 3. Both yoga and massage helped their back pain 4. Both yoga and massage helped some aspects of their back pain 5. Neither yoga or massage helped their back pain 6. Insufficient data was collected to make a conclusion

This article contains several examples of participant reports. Figure 1 shows example participant reports with written information to show pain comparisons for yoga versus usual care and massage versus usual care. Six potential displays show various levels of effectiveness of massage and yoga treatments, ranging from both treatments helping pain to neither helping pain with a final potential display created for participants who had insufficient data to identify the effectiveness of massage or yoga treatment. Figure A1 shows a visual display comparing daily pain patterns (assessed using the PROMIS scales), side effects, and pain medication use during each of the treatment phases. Figure A2 provides visual summaries of EMA measures of pain, fatigue, and stress and comparisons of these measures between treatment phases 24 hours following treatment. Figure A3 shows two example displays of adherence: one with high adherence to study measures and one with low adherence. The report also included a page offering a summary of the overall study results and a lay explanation of statistical significance (Figure A4). Including the summary page and a title page, each report was 10 pages in length and covered results for all clinical outcomes measures used in the trial.

Based on the literature available at the time and the intent to deliver the reports virtually to study participants, the design of the participant report aimed to provide information that would resonate with various information display styles and engagement interest levels. We provided written summary components for participants who preferred to read through their results. The researchers used graphs and figures to display results for visual learners. In addition, the study team developed a library of visual icons (e.g., an icon of a Fitbit device) to supplement numeric and written results. Finally, the researchers included links to brief video explanations, generated using animated and annotated visuals of a sample report with explanatory audio voiceover from a research coordinator, on every result page to account for participants who preferred a more auditory experience. The researchers decided to use statistical significance throughout the report to maintain the scientific integrity behind personalized trials while prioritizing that participants received meaningful and clear results. Participants had the opportunity to schedule a phone call with the study team to discuss their report or to ask any questions they may have had about the findings illustrated in their report.

The researchers prioritized several considerations when deciding which elements to present in these participant reports and how to present them. Based upon lessons learned from previous piloting activities (Kronish et al., 2019), priorities consisted of reporting observed data directly back to the participant with minimal interpretation and ease of comprehension (so those not accustomed to seeing their data this way could easily understand their results). In addition, the research team only included actionable insights to facilitate integration of trial/report learnings into participants’ future wellness habits. For example, if individuals found that yoga treatment reduced pain and fatigue, they could incorporate yoga treatment into their pain management regimen. The reports avoided providing medical advice/diagnoses since they were focused on a general audience rather than clinicians.

The participant report included multiple visual characteristics to support participant comprehension. These visual characteristics included clear, differentiated colors to represent the three treatment blocks: blue icons represented yoga, purple icons represented massage, and orange icons represented usual care. The researchers used colors and other visual cues to illustrate directions of change. For example, a red arrow pointing downward represented a decrease in step count, while a red ‘X’ represented an increase in self-reported momentary stress levels. Conversely, a green arrow pointing upward represented an increase in sleep duration, while a green checkmark represented a decrease in self-reported momentary pain levels. When possible, researchers used simple icons and visual arrangements in place of complex graphs and charts to minimize unnecessary participant confusion or intimidation. Finally, the intentional incorporation of white space, although leading to a longer report, aimed to focus participants on key insights without overwhelming them with data on each page.

The study team manually constructed a report for each participant using an institutional review board (IRB)–approved template created in Microsoft PowerPoint. The template contained static language in each report that the research team updated based on statistical analysis and supplemental data findings. Once the statistician completed the data analysis (described in (n.d.-l) “An R Shiny App for a Chronic Lower Back Pain Study, Personalized N-of-1 Trial,” also included in this special issue), the statistician sent an HTML file containing average estimates and *p* values for each clinical outcome by treatment to the study team. In addition, to average estimates over the treatment period, the statistical analysis also contained estimates and *p* values for increases in the above categories one day after massage and one day after yoga. Research team members then entered values from the statistician into the participant report. To provide for a more comprehensive participant report, the researchers supplemented the statistician report with simple statistical analyses generated using data from the Fitbit device and participant survey responses. The research team manually constructed and formatted written summary details of the participant report. The primary pain outcomes (i.e., pain intensity and pain interference) determined overall conclusions (i.e., “Massage helped your back pain best”). The research team included other secondary outcomes (i.e., side effects and medication intake) when relevant (i.e., “we observed several positive health benefits during yoga weeks that may be of interest to you”). Several team members reviewed each report to ensure accuracy prior to distribution to participants. Finally, the study team converted each PowerPoint file to a PDF file before sending the report to the participant. A research coordinator sent the participant report via encrypted email to the participant’s internal organization email address to ensure security.

### 2.3. Satisfaction With the Feedback Reports

Researchers obtained participant satisfaction on the feedback report via REDCap^®^ survey. Five questions assessed participant satisfaction with their personalized feedback report. For example, participants rated their agreement or disagreement with statements such as “I found the data report easy to understand” and “I found the data report useful for understanding how my chronic lower back pain affects me.” Researchers also assessed participant satisfaction with the interventions (both massage and yoga) and with the Zeel^™^ platform via the emailed survey. Participant ratings ranged from 1 to 5 as a representation of a Likert scale. Depending on the questions, a value of 1 signified either “Not at all Satisfied” or “Strongly Disagree,” while a response of 5 was either “Very Satisfied” or “Strongly Agree.” Results for the satisfaction questions were represented using means and standard deviations calculated using R, version 4.0.4 (n.d.-m).

### 2.4. Follow-Up Interviews

Following the completion of the participant report satisfaction survey, research coordinators invited participants to partake in a 45-minute, semi-structured follow-up interview via Microsoft Teams or phone call to offer additional qualitative feedback on the report that they had received. Research coordinators asked IRB-approved questions spanning various study themes and recorded qualitative data using an internal REDCap^®^ survey. Seven of the follow-up interview questions related to the participant feedback report. These questions obtained qualitative feedback on general report comprehension and visualizations, perceived gaps in reported findings, and future plans for pain management and wellness habits based on report learnings.

## 3. Results

**Figure 2.**
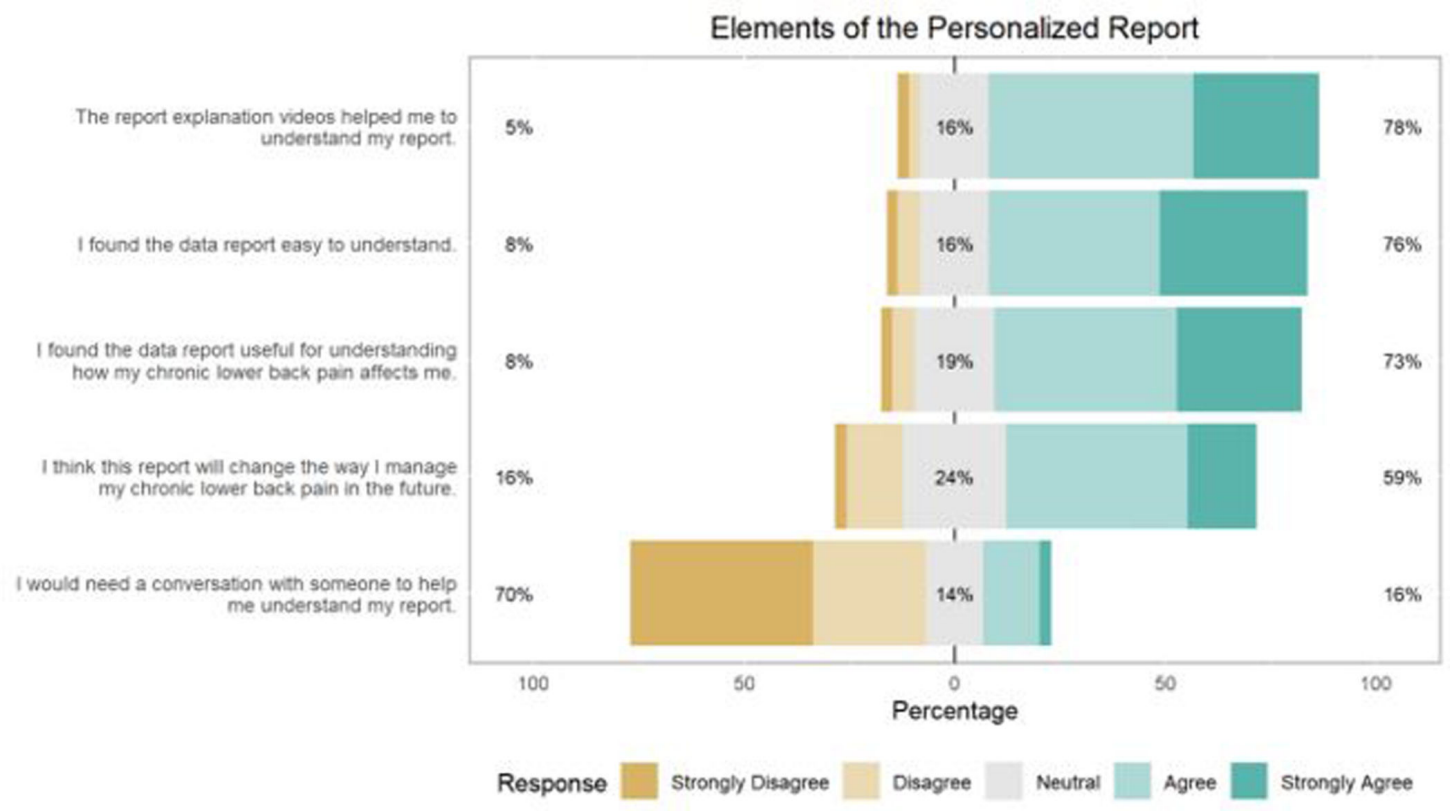
Participant satisfaction with elements of the personalized report (*N* = 37). *Note.* The shades of brown reflect the varying degrees of disagreement to the questions. The shades of blue reflect the differing degrees of agreement. The grey color is for a neutral response.

The study enrolled 57 participants in the trial. Two individuals requested to discontinue the intervention and be withdrawn from the study for personal reasons impacting their ability to complete the study protocol. Of the 55 participants who completed the trial, 37 (62%) completed the satisfaction survey. Figure 2 displays participant responses to the questions in the satisfaction survey. Overall, participants responded positively to elements of the personalized reports, with mean scores ranging from 3.6 to 4.0 on the first four questions. The last question assessing satisfaction with the personalized report (“I would need a conversation with someone to help me understand my report”) had a mean score of 2.1, indicating that participants did not generally feel they would need help in interpreting the information presented in the personalized report. Therefore, most participants stated that the personalized reports were easy to understand, helpful with understanding how CLBP affected them, and significant in helping them determine how to best manage their own CLBP. When participants evaluated how they felt about the presentation of their results through the personalized reports, participants stated their satisfaction with an average response score of 4.162 (Table 1). Figure 2 contains the distribution of the five questions assessing satisfaction with the personalized report, showing that most of the participants responded positively to all questions. Approximately 78% (*N* = 29) of the participants responded that the explanation videos helped them to understand their report. Over 70% (*N* = 28) of the participants stated that understanding the report was easy and that they found their personalized report useful for understanding the way CLBP affected them (*N* = 27). In addition, approximately 70% (*N* = 26) of the participants stated that they did not need to talk with a coordinator to get help with understanding their report, implying that most participants considered the report and the explanation videos to be adequate for their understanding. More than half (59%, *N* = 22) of the participants agreed that the information provided in their personalized reports may change the way they manage CLBP. However, a number of participants (24%, *N* = 9) did remain neutral in their response to this question.

Fifteen participants (25%) completed a follow-up interview after administration of the satisfaction survey. Most respondents stated that they found the participant reports easy to understand and enjoyable to use. Several participants reported issues accessing their encrypted participant report; some participants reported poor usability of the web interface required to open the encrypted email (e.g., the participant could not log in or logged in but did not see the attachment). Furthermore, participants requested that their personalized reports be re-sent due to expired access of the link to view the encrypted email. Individuals who reported difficulties with the contents of the report cited the amount of information provided and the time needed to read the report as being primary barriers to comprehension. Although participants found the video summaries useful, some individuals reported feeling confused when the video discussed results in a different order than the report. During the follow-up interview, most participants stated that the report would be helpful for managing their back pain. One participant stated that, though they initially preferred massage, they considered practicing yoga more frequently after viewing their report, which contained data showing how yoga was more effective for their pain reduction. Other participants stated that they did not believe the report was useful for managing their back pain.

## 4. Discussion

The findings from the current examination of participant feedback reports suggest that the personalized feedback reports developed for this trial conform with recommendations for lay summaries of trial results and incorporate important considerations from the patient education literature. The reports incorporated key elements of the treatment, discussed the condition of CLBP, showed personal results, displayed adverse events (such as side effects), and provided a summary of how the trial results may help participants to manage their CLBP in the future. The presentation of the reports used written, visual, and auditory formats to provide information in an easily understandable manner that was suitable to most adults.

The results from the satisfaction survey administered in the current study show that participants were generally satisfied with their personalized feedback report. The majority of participants (>70%) found the report easy to understand, the data in the report useful, and the accompanying video explanation helpful. Furthermore, 70% of participants did not feel they needed a conversation with a research team member to explain the contents of their report. In fact, although the study team provided all participants the option of a one-on-one meeting to discuss their report results, no participants elected to have the meeting. This indicates that a large proportion of participants found the report to be useful and easily understandable. Importantly, it also supports the potential scalability of delivery of personalized trial reports directly to research participants without the need for further involvement of the study team or a physician.

Interestingly, a majority (59%) of the sample felt the personalized report would help to change the way they managed their CLBP. Therefore, while most individuals expressed satisfaction with the personalized feedback report, 40% of the sample did not feel the information they received would change how they deal with back pain. One individual expressed the belief that their experience over the 14-week trial provided a better indication of how to continue managing pain than a report itself. However, given the heterogeneity of treatment response (HTE) typically seen in personalized trials, different participants’ varying opinions on the report based on their personal responses to the treatment are to be expected. For example, if an individual experienced a similar decrease in CLBP with both massage and yoga but experienced better sleep and additional stress reduction benefits with yoga only, that participant’s data report may be more influential in assisting his or her pain management strategy. Additionally, several participants stated that the volume of information in the report was confusing or that they preferred to manage their pain on their own. These perspectives potentially provide some insight into why the feedback reports were not useful for some participants. Additional research and pilot-testing will be required to examine how we can increase the utility of feedback reports for participants while decreasing the amount of information presented. Future activities might entail asking participants if they prefer auditory, visual, or written descriptions and only sending the information in their preferred format for future reports.

In the process of developing the participant reports for the current study, the investigators identified several problems and potential areas for improvement. A major limitation of the study and for the ultimate adoption and scalability of personalized trial feedback reports was the amount of effort required to generate each report. For the current trial, research team members generated participant reports one at a time using information from statistician reports. This process contributed to a 3-month delay in providing the reports to the participants after they concluded their personalized trial. The endeavor required a substantial time investment from the research team and involved analyzing large volumes of information, crafting statistical results into text summaries and visual displays, and manually formatting these elements in a Microsoft PowerPoint file. It is possible the delay between the personalized trial end date and participants receiving their results report may have negatively impacted survey response rate and the participants’ ability to describe the usefulness of the reports.

To address this limitation, we are currently exploring methods to automate the process of participant report generation for future studies. The study team has tested multiple platforms and programs to reduce latency time between data collection and personalized report delivery. As a result of this exploration, the team selected R Markdown as the best file format to generate the PDF participant reports for future personalized trials; it allows for writing and executing code for data analysis within the actual report document. Using R Markdown, our research team is developing methods to extract participant data, conduct statistical analyses, and input parameter estimates from these analyses (including estimates of treatment effects and *p* values for statistical analyses) directly into a participant report. With R Markdown’s PDF document, users are able to access readily available document-formatting options, such as styling text that includes mathematical operations. These features will allow members of the research team to properly format the participant reports and incorporate the same level of written and visual information within the reports as those that had been manually generated by the study team. The low cost of using R Markdown to generate PDF files also contributed to the decision to use R Markdown for future personalized participant reports. In addition, R Markdown also has the flexibility to output reports in additional formats including HTML, Microsoft Word, and Microsoft PowerPoint. The research team intends to fully automate feedback report generation for personalized trials, thereby increasing the utility of the current personalized design and reducing overall costs. Appendix B, “Utilizing R Markdown to Automate Participant Feedback Reports,” includes additional description of the planned automated report-generation process using R Markdown.

As the researchers collect additional data and better understand a minimal viable product for personalized participant reports, the study team will replace R Markdown with custom web application programming focused on user experience and user design. Future studies will use a secured online portal to disseminate feedback reports in place of a PDF file that is distributed via encrypted email. Some participants had difficulty accessing information using encrypted email or waited too long to view their reports, causing their access link to expire. A secure online portal would provide participants with the ability to access their research results on their own time while reducing potential technological complications. In addition to increasing ease of access, a custom report application via an online portal may reduce participants’ feeling overwhelmed by allowing them to filter and view the results elements that specifically interest them rather than placing those results in the context of a large report.

The current study represents a successful implementation of a participant feedback report for a personalized trial design examining the effects of massage and yoga treatment for CLBP. These feedback reports provided trial participants with their individual results in a clear, understandable manner and offered guidance for utilizing the information to help with management of CLBP symptoms. The researchers will make additional modifications to fully automate the report-generation process, allow for easier access to report results, and refine the presentation of the report to increase participant comprehension, scalability, and usability of the report in future personalized trials.

## Supplementary Material

Supplemental Figure 1

Supplemental Figure 2

Supplemental Figure 3

Supplemental Figure 4

1

## Figures and Tables

**Table 1:** Descriptive Statistics for Satisfaction Measures for the Personalized Report and Personalized Trial (N=37).

Measure		Mean (*SD*)	Range
Elements of the Personalized Report*			
Items	The report explanation videos helped me to understand my report.	4.0 (0.913)	[1, 5]
	I found the data report easy to understand.	4.0 (1.0)	[1, 5]
	I found the data report useful for understanding how my chronic lower back pain affects me.	3.919 (0.983)	[1, 5]
	I think this report will change the way I manage my chronic lower back pain in the future.	3.568 (1.015)	[1, 5]
	I would need a conversation with someone to help me understand my report.	2.054 (1.177)	[1, 5]

Questions were rated on a 5-point Likert scale from 1 “Strongly Disagree” to 5 “Strongly Agree.”
