# Supplementary figures and images for: Personalized Feedback for Personalized Trials: Construction of Summary Reports for Participants in a Series of Personalized Trials for Chronic Lower Back Pain
Source: Harv Data Sci Rev. Author manuscript; Available in PMC 2023 Nov 24. (PMC10673635; doi:10.1162/99608f92.d5b57784)

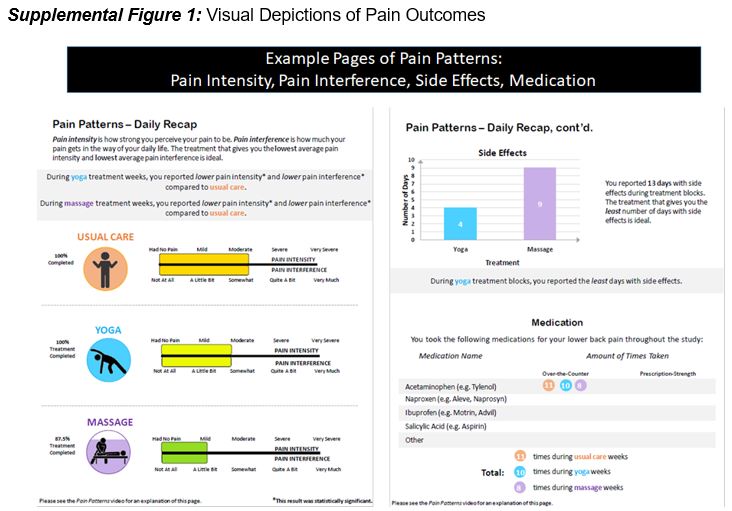

Supplement: Supplemental Figure 1 [file NIHMS1882398-supplement-Supplemental_Figure_1.jpg]

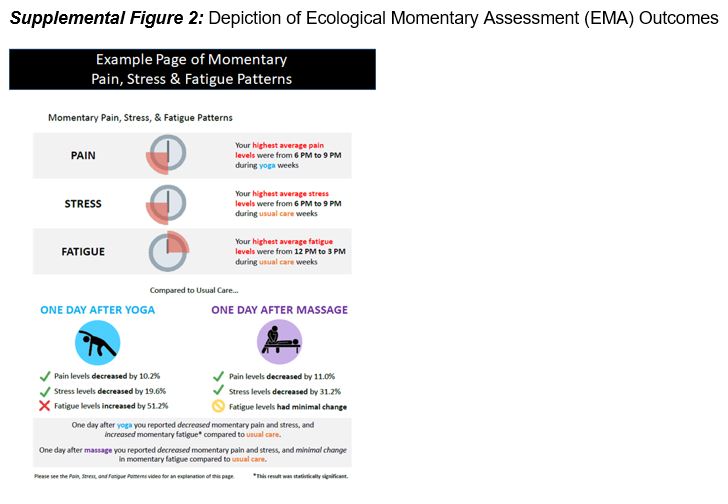

Supplement: Supplemental Figure 2 [file NIHMS1882398-supplement-Supplemental_Figure_2.jpg]

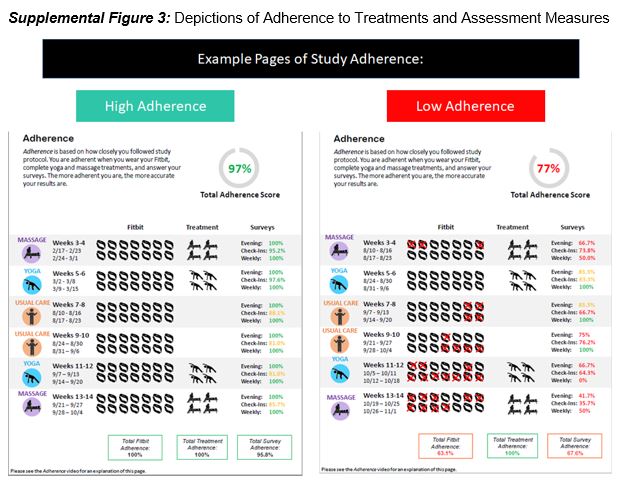

Supplement: Supplemental Figure 3 [file NIHMS1882398-supplement-Supplemental_Figure_3.jpg]

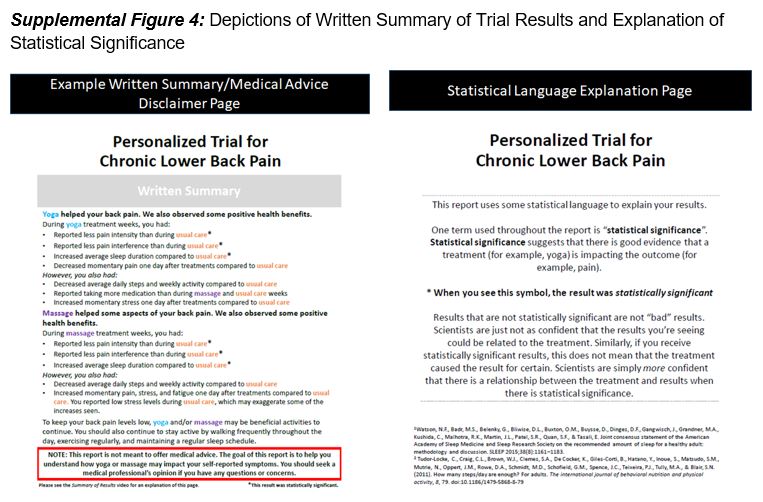

Supplement: Supplemental Figure 4 [file NIHMS1882398-supplement-Supplemental_Figure_4.jpg]
