# Supplementary material for: Personalized Feedback for Personalized Trials: Construction of Summary Reports for Participants in a Series of Personalized Trials for Chronic Lower Back Pain
Source: Harv Data Sci Rev. Author manuscript; Available in PMC 2023 Nov 24. (PMC10673635; doi:10.1162/99608f92.d5b57784)
Supplement: 1 [file NIHMS1882398-supplement-1.pdf]

Appendices

Appendix A. Supplementary Figures

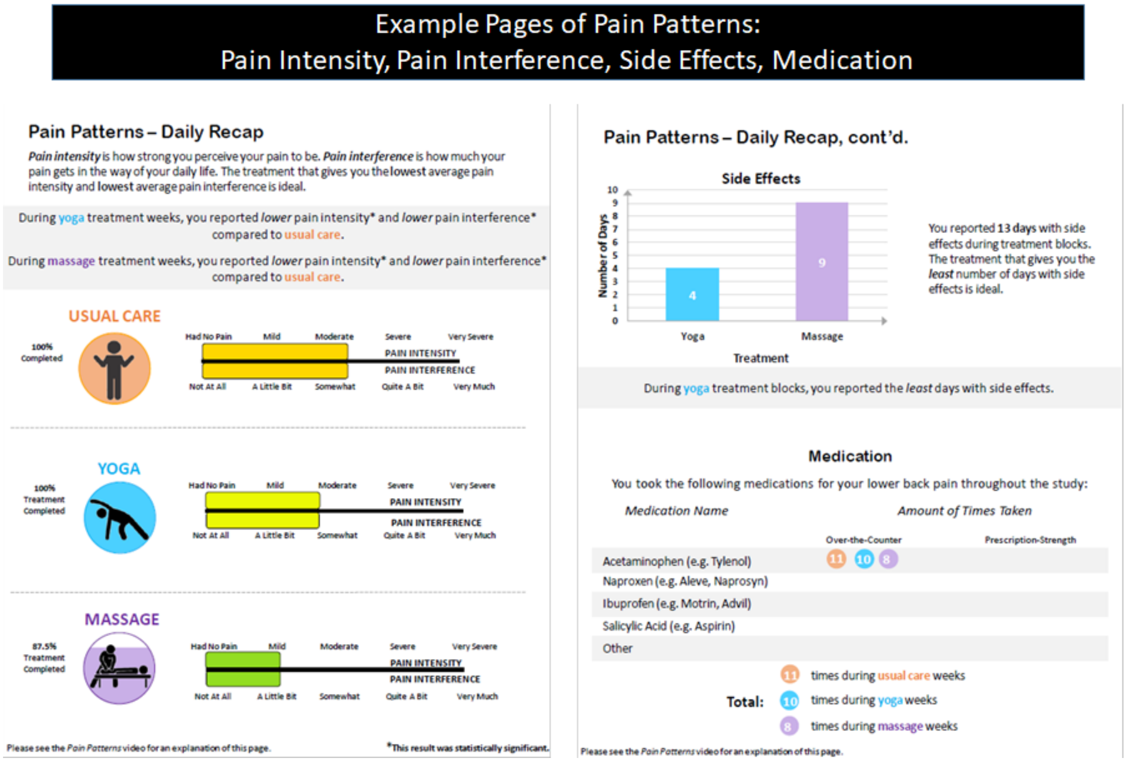

Figure A1. Visual depictions of pain outcomes.

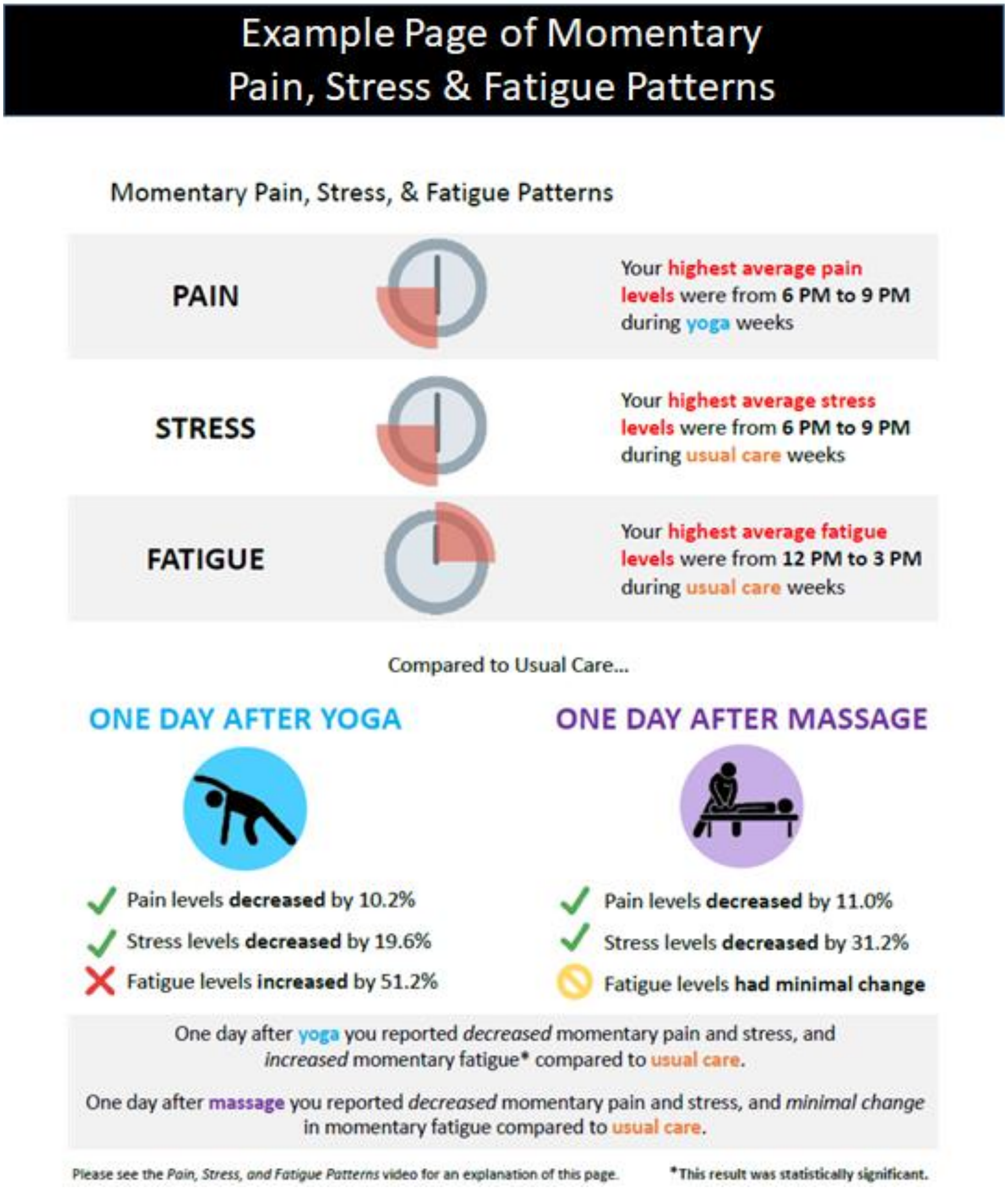

Figure A2. Depiction of ecological momentary assessment (EMA) outcomes.

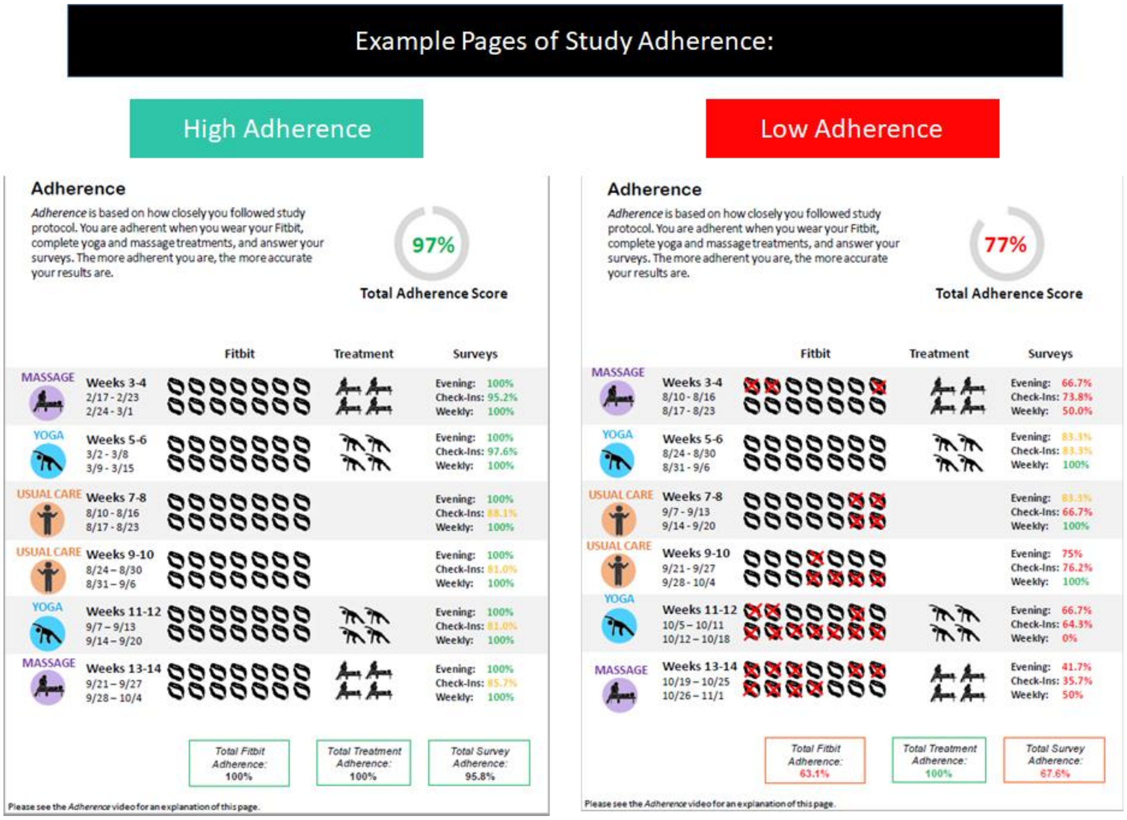

Figure A3. Depictions of adherence to treatments and assessment measures.

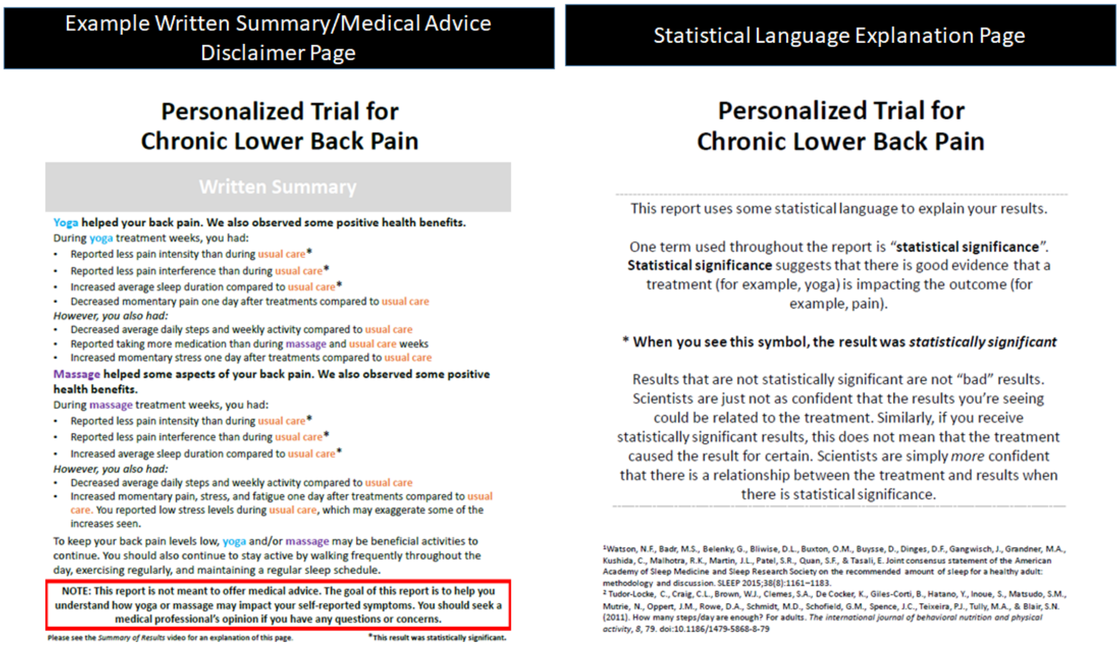

Figure A4. Depictions of written summary of trial results and explanation of statistical significance.

Appendix B. Utilizing R Markdown to Automate Participant Feedback Reports

This section elaborates on the process of utilizing R Markdown to generate automated participant feedback reports for personalized trials. R Markdown is a strong report generation tool since it provides users with various output format options. The default output document file formats include HTML, PDF, and DOCX (i.e., Word). Other output formats are also readily available in the presentation slide styles such as HTML and PowerPoint. As the participant reports for personalized trials need to be in easily accessible document format, we selected the PDF document output. R Markdown's PDF allows users to access readily available document formatting options such as styling text, including mathematical operations, and altering figure heights and alignments on the page. To be able to use R Markdown, the programming language R (n.d.-n) and the integrated development environment (IDE) RStudio need to be installed. Everything can be downloaded for free using the Open-Source Edition of RStudio. R Markdown comes with RStudio, but alternatively, the package "rmarkdown" (n.d.-o) can be installed and loaded in the code to render the PDF files in a typical R code file separately. To be able to generate the PDF reports using R Markdown, a form of LaTeX (n.d.-p), a document preparation system, is used.

The output files from R Markdown consist of three main structural components: metadata, text, and code. The metadata is written with the syntax YAML (YAML Ain't Markup Language; (n.d.-q)). Text is written with the syntax used for typical R Markdown files. The code can be utilized in R Markdown files in two different ways. The first is called 'code chunks,' which allow users to write several lines of executable code in an easy-to-read format that offers easy access to the code for editing. These code chunks also provide various options for the user to help determine what gets outputted onto the reports. One such example is the option of either displaying or hiding the code in the output file. Hiding the code chunks executes the code but does not print the actual code when the output files are rendered. Plenty of other options, such as hiding extraneous messages and comments or setting the alignment of the figures on a page, are available. The second method is called the 'inline code,' which allows users to run R code within the text portion of the document. Many use cases of inline code are to reference code results from a code chunk directly into the text so that it displays only the object from the code. This also helps to apply text formatting tools (e.g., text color, bolding of the text) to the code results (n.d.-r).

Through the usage of code in R Markdown documents, graphs, images, and tables can also easily be incorporated and formatted into the output PDF document in a clean manner. Packages such as "grid" (n.d.-s) and "gridExtra" (n.d.-t) are available to help with the design of the report pages. With these packages, designing pages to appear like those displayed in the adherence pages of Figure A.3 is possible. To design such pages, the icon image files are read, and relevant data can be transformed to display particular images or colors based on certain values and subsequently inserted into a table/grid format while incorporating icons to highlight the data. The various design capabilities provide document outputs such as PDFs and, therefore, offer multiple possibilities to help in designing the reports.

Automation of these reports can be executed through separate R code using the render function that comes with the "rmarkdown" (Baumer & Udwin, 2015) package in R;

beforehand, all the text and code is set up with R Markdown as a template. Once the template is prepared, the next step is to set parameters in the metadata portion of the R Markdown documents; the code grabs the parameters and a specific participant's data from a large data set in order to generate the report. Outside of the R Markdown file, a separate R code file could be written to use the render function, where a written code is then used to generate a report for each of the participants of the study by iterating through each record ID. Automating the reports, therefore, removes the intensive manual work and time that were required in the Chronic Lower Back Pain reports. This method will significantly reduce the requisite time in which to manually generate the reports for each participant; what would normally require multiple days or even weeks through Microsoft PowerPoint can now take a single day.

Appendices References

Auguie, B., Antonov, A., & Auguie, M. B. (2017). Package "gridExtra." *Miscellaneous Functions for "Grid" Graphics*. https://cran.r-project.org/web/packages/gridExtra/gridExtra.pdf

Baumer, B., & Udwin, D. (2015). R markdown. *Wiley Interdisciplinary Reviews: Computational Statistics, 7*(3), 168–177. https://doi.org/10.1002/wics.1348

Ben-Kiki, O., Evans, C., döt Net, I. (2009). *YAML Ain't Markum Language (YAML™) Version 1.2*. https://yaml.org

Murrell, P. (2002). The grid graphics package. *R News, 2*(2), 14–19. https://www.r-project.org/doc/Rnews/Rnews_2002-2.pdf

R Core Team. (2013). *R: A language and environment for statistical computing*. R Foundation for Statistical Computing. http://www.R-project.org/

The LaTeX Project. (n.d.) Retrieved from https://www.latex-project.org

Xie, Y., Allaire, J. J., & Grolemund, G. (2018). *R markdown: The definitive guide*. CRC Press.
